# A finite element study on three maxillary protraction devices for treating maxillary sagittal hypoplasia at different levels of bone fusion

**DOI:** 10.3389/fbioe.2026.1745618

**Published:** 2026-02-11

**Authors:** Kaiyuan Sun, Gang Zhao, Yinan Jin, Bowen Tang, Yufei Liu, Lu Wang

**Affiliations:** 1 Stomatology Collage of Jiamusi University, Jiamusi, Heilongjiang, China; 2 Department of Orthodontics, Affiliated Stomatological Hospital of Jiamusi University, Jiamusi, Heilongjiang, China

**Keywords:** bone anchorage, finite element analysis, invisible aligners, maxillary protraction, maxillary suture, micro-implants

## Abstract

**Objective:**

This study utilized three-dimensional finite element analysis to assess stress distribution in the maxilla and sutures, as well as craniofacial bone and tooth displacement, in patients exhibiting mild to moderate sutural fusion when using three distinct maxillary protraction devices.

**Methods:**

A craniofacial composite model with mild to moderate sutural fusion was constructed and fitted with three types of appliances: a tooth-supported maxillary protraction device, a micro-implant maxillary protraction device, and an invisible aligner maxillary protraction device. A traction force was applied at a 30° forward-downward angle relative to the occlusal plane. The equivalent elastic strain conditions of the maxilla and suture lines were compared and analyzed, along with craniofacial bone and tooth displacement under various loading conditions.

**Results:**

Stress distribution in the sutures during maxillary protraction varied among the three appliance types. The micro-implant group exhibited the highest equivalent strain in the frontal-maxillary and naso-maxillary sutures, while the zygomatic-temporal suture experienced the greatest strain in the clear aligner group. Under the same level of suture fusion, the clear aligner group displayed the most significant maxillary displacement and incisor labial inclination, in contrast to the micro-implant group, which showed the least incisor labial inclination. As sutural fusion increased, both the stress on the sutures and the extent of skeletal displacement significantly decreased, while the propensity for maxillary rotation increased.

**Conclusion:**

Each of the three appliance types effectively facilitates maxillary advancement, though they result in varying degrees of incisor labial inclination. The osteogenic effect of maxillary protraction diminishes with increased sutural fusion. The micro-implant maxillary protraction appliance is advisable for patients with nasal root depression, while the clear aligner variant is preferable for those with midface depression. Furthermore, in patients with advanced sutural fusion, it is crucial to adjust the traction direction to mitigate undesirable maxillary rotation.

## 1 Introduction

Skeletal Class III malocclusion is a craniofacial deformity resulting from genetic and environmental factors that disrupt normal jaw development. Clinically, it is primarily characterized by maxillary underdevelopment, mandibular overgrowth, or a combination of both. Studies indicate that approximately half of patients with skeletal Class III malocclusion present with maxillary hypoplasia. (Ellis and McNamara, 1984; Kathem and Pedersen, 2025; Rutili et al., 2023; Wang et al., 2022a). Children with maxillary sagittal hypoplasia frequently present with a depressed midface and nasal root. If not addressed through early orthodontic intervention, this condition can profoundly affect a child’s physical and mental wellbeing. For cases of mild to moderate maxillary sagittal hypoplasia, maxillary anterior traction is often utilized as an orthopedic treatment (Kathem and Pedersen, 2025; Cho et al., 2025). This technique applies traction forces to the sutures, stimulating anterior and inferior growth of the maxilla. The maxilla is interconnected with other facial bones via the maxillary sutures. Research indicates that the success of maxillary advancement during protraction is contingent upon the extent of suture fusion (Angelieri et al., 2017a; Li et al., 2023). While the fusion degree of the maxillary sutures is crucial for the effectiveness of protraction, existing studies on suture biomechanics have largely focused on a singular fusion stage. Investigations into maxillary protraction have mainly emphasized whether it involves arch expansion and the design of appliances, with limited exploration into the biomechanics of the craniofacial complex as the degree of suture fusion varies. The zygomatic bone connects to the maxilla through the zygomaticomaxillary suture and to the temporal bone via the zygomaticotemporal suture. Recent studies (Vracar et al., 2021) show that maxillary anterior traction can lead to an anterior and inferior shift of the zygomatic bone, a change that carries significant implications for facial aesthetics. The height and prominence of the cheekbones directly influence the three-dimensional contour of the face. Higher cheekbones enhance the layered appearance of the midface, thereby improving the overall harmony of facial structure. Additionally, current research (Wang et al., 2022b; He and Wu, 2025) highlights the critical role of the frontonasal suture in the development of the middle third of the pediatric face. Thus, comprehending the stress distribution within the maxillary sutures during protraction can provide valuable insights into predicting post-treatment enhancements in facial aesthetics.

As the most commonly employed traction device in clinical orthodontics, the tooth-supported maxillary protraction appliance has proven its effectiveness in treatment outcomes. However, its application can lead to dental compensatory changes, such as the labial inclination of maxillary anterior teeth (Zhao et al., 2023). In contrast, bone-anchored maxillary protraction devices apply traction forces directly to the maxilla, resulting in more pronounced skeletal effects (Si et al., 2023). Nevertheless, the invasive nature of these devices can deter some families of pediatric patients from choosing them. Recent research (Blevins, 2019; Ma et al., 2024) indicates that clear aligners can be effectively combined with maxillary protraction devices for treating adolescent patients. Unlike traditional intraoral appliances, which may induce significant discomfort and yield inconsistent results, clear aligners are known for their enhanced comfort. However, the biomechanical implications of using clear aligners alongside maxillary protraction devices on the craniofacial complex remain to be fully understood.

Medical finite element analysis (FEA) is a computational technique used to evaluate the stress characteristics of biological structures. Its non-invasive nature and numerous advantages have made it a popular choice for exploring complex mechanical issues in orthodontics (Zhang and Liu, 2023; Zhang et al., 2025b). In this study, three-dimensional FEA was utilized to create models representing mild and moderate maxillary suture fusion. This approach allowed for the quantification of how varying degrees of suture fusion impact craniofacial bones, sutures, and teeth during the process of maxillary protraction. The findings contribute biomechanical insights essential for tailoring treatment plans for patients with skeletal Class III malocclusion across different growth and developmental stages.

## 2 Materials and methods

### 2.1 Equipment and software

For this study, 64-slice spiral CT imaging was used to scan the craniofacial regions of volunteers, encompassing the area from the inferior margin of the sixth cervical vertebra to the upper third of the forehead. Three software applications—Mimics 21.0, Geomagic Wrap 2021, and SolidWorks 2021—were utilized to create and assemble three-dimensional models of the craniofacial bones, periodontal ligaments, maxillary teeth, sutures, and orthodontic appliances. Biomechanical simulations were subsequently conducted using Ansys Workbench 2022 R2 software.

### 2.2 Research subjects

A 12-year-old patient diagnosed with Class III maxillary hypoplasia was selected for this study. At the early stage of permanent dentition, the maxillary teeth numbered 16–26 had erupted. Based on the classification method for suture fusion degrees proposed by Angelieri et al. (2017b) and the cervical bone age staging method proposed by McNamara (McNamara and Franchi, 2018), this patient was assessed to be at zygomatic-maxillary suture stage B, while the cervical bone age was determined to be CS2. The angles measured were as follows: sella-nasion A point angle at 76.6°, sella-nasion B point angle at 79.1°, and the angle formed by the sella-nasion A point, nasal root point, and sella-nasion B point was −2.5°. The patient had no prior orthodontic treatments or systemic diseases influencing craniofacial development.

### 2.3 Model construction

**FIGURE 1:**
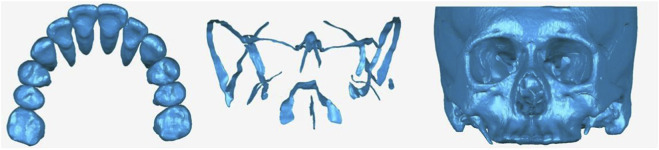
Three-dimensional models of teeth, craniofacial bones and maxillary sutures.

CT scan data were imported into Mimics software to create an initial three-dimensional model of the craniofacial complex. This model was then transferred to SolidWorks software, where cranial sutures were constructed, including the frontal-maxillary, nasal-maxillary, median palatal, bilateral zygomatic-temporal, zygomatic-frontal, zygomatic-maxillary, zygomatic-sphenoid, and pterygopalatine sutures. The thickness of the sutures was set at 0.5 mm. Optimization was conducted using Geomagic software, followed by the creation of a 0.25 mm thick linear elastic periodontal ligament. Finally, the craniofacial bones, periodontal ligament, teeth, and sutures were assembled to form the experimental model (Figure 1).

**FIGURE 2:**
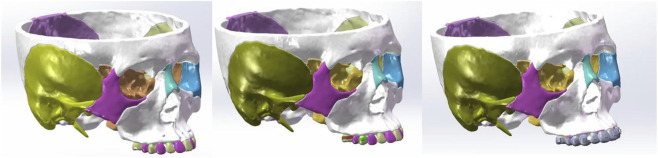
3D model assembled with maxillofacial complex and three maxillary protraction devices.

Using SolidWorks 2021, three orthodontic appliance models were developed. The tooth-supported anterior traction device model was constructed based on the actual design and dimensions of clinical appliances. Orthodontic bands (3 mm wide, 0.2 mm thick) were placed bilaterally on the first premolars and first molars, and connecting rods with a diameter of 1.2 mm integrated the appliance into a single unit. Traction hooks extended from the buccal bands of the first premolars to the gingival margin of the canines. For the micro-implant anterior traction device, microimplants were simplified as cylindrical structures (3 mm in diameter and 7 mm in length). Following clinical practice (Wang et al., 2021), they were positioned between the roots of the maxillary canines and lateral incisors, 8 mm from the gingival margin. The invisible aligner model (0.5 mm thick) was constructed on the crown surfaces without brackets (Fan et al., 2023; Cengiz et al., 2025). Angled hooks (Ma et al., 2024) were placed on the mesial surfaces of both canines. To prevent slippage or detachment during testing, vertical rectangular attachments (3 × 2 × 1 mm) (Li et al., 2025a) were designed on the anterior teeth and molars. These attachments served solely for retention and to facilitate tooth movement. Finally, the three appliance models were integrated with the three-dimensional craniofacial complex model to create the final assembly (Figure 2). Based on the degree of suture fusion and the type of aligner device, six working conditions were established for the simulation experiments (Table 1).

**TABLE 1:** Arrangement of the experimental group.

Model number	Degree of bone fusion	Appliance
A1	Mild fusion	Micro-implant device
A2	Moderate fusion	Micro-implant device
B1	Mild fusion	Tooth-supported device
B2	Moderate fusion	Tooth-supported device
C1	Mild fusion	Clear aligners
C2	Moderate fusion	Clear aligners

### 2.4 Material properties, boundary conditions, contact settings, and applied loads

Assuming that all materials used in this experiment are isotropic, homogeneous, continuous linear elastic materials, the elastic moduli and Poisson’s ratios for each structure have been established based on previous literature (Shyagali et al., 2023; Shi et al., 2020; Liu et al., 2022) (Table 2). The craniofacial composite model was anchored at the foramen magnum and forehead with zero-displacement and zero-rotation constraints (Han et al., 2024; Garg et al., 2022) to eliminate any unnecessary movement. Following prior research (Akdemir and Gorucu-Coskuner, 2024; Bocklet et al., 2024), binding contacts were created between cortical bone and sutures, teeth and periodontal ligaments, periodontal ligaments and maxillary bone, microimplants and maxillary bone, orthodontic bands and teeth, as well as traction hooks and invisible aligners. Adjacent teeth were treated as non-separable, while the invisible aligners and attachments were configured as friction contacts with the outer surfaces of the tooth crowns, using a friction coefficient of 0.2 (Zhu et al., 2024; Li et al., 2025b). The assembled model was imported into ANSYS Workbench 21.0 for meshing. The micro-implant group consisted of a total of 749,405 meshes, the dental abutment group contained 759,868 meshed elements, and the invisible aligner group had 779,943 meshed elements. Consistent with previous studies (Choi et al., 2023; Liu, 2024), a traction force of 5 N was applied per side at the bilateral traction sites for each model group, directed downward at a 30° angle relative to the occlusal plane.

**TABLE 2:** Properties of each material in finite element models.

Material	Young’s modulus (MPa)	Poisson’s ratio
Bone	13,700	0.3
Mild fusion of sutures	70	0.4
Moderate fusion of sutures	500	0.4
Tooth	20,290	0.3
Periodontal membrane	0.68	0.45
Orthodontic miniscrew	110,000	0.3
Orthodontic bands	190,000	0.33
Steel	200,000	0.33
Invisible aligner	528	0.36
Angel button	114,000	0.35
Attachment	12,600	0.37

### 2.5 Establishment of the coordinate system

A 3D coordinate system was established at the center of the maxilla. In this system, the X-axis represents the horizontal direction, with positive values extending to the right side of the model. The Y-axis indicates the sagittal direction, with positive values moving forward. The Z-axis denotes the vertical direction, perpendicular to the orbital-temporal plane, with positive values directed upward. This unified coordinate system is consistently utilized throughout the study to describe tooth displacement patterns. The positive directions of the X, Y, and Z-axes correspond to distinct displacement trends: incisors displace distally, labially, and downward; first molars displace buccally, mesially, and downward.

### 2.6 Observation indicators

This study analyzes the following structures:(a) Evaluates the equivalent elastic strain of the following five sutures: Zygomaticomaxillary suture, zygomatic temporal suture, frontalmaxillary and nasofrontal suture, pterygopalatine suture, and median palatal suture; (b) Evaluation assessment of equivalent stress distribution within the maxilla, also measured in MPa; (c) the quantification of displacement at specific craniofacial skeletal landmark nodes, measured in μm: including the nasal root point (N), anterior nasal spine (ANS), posterior nasal spine (PNS), alveolar base point (A), maxillary central incisor point (U1), midpoint of the orbital rim (Or), and zygomatic body (JU); (d) Evaluation of maxillary incisor and molar displacement, in μm: utilizing reference points such as the midpoint of the incisal edge of the central incisor (a1), midpoint of the incisal edge of the lateral incisor (a2), and the mesial buccal cusp of the first molar (a6). The study employs a symmetrical default model, focusing on the right side for analysis. As no horizontal forces were applied during the experiment, only sagittal and vertical displacements at the defined reference points were recorded.

## 3 Results

### 3.1 Force distribution at bone joints in each model group

**FIGURE 3:**
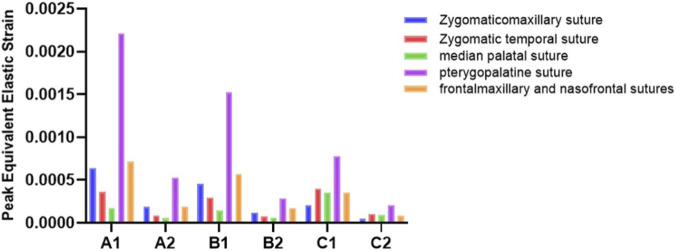
Peak equivalent elastic strain in the five maxillary sutures under loading across different models.

**FIGURE 4:**
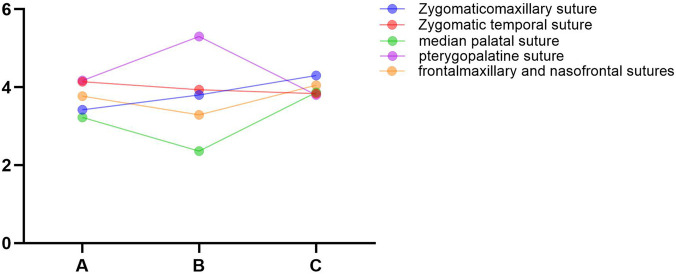
Ratio of the peak equivalent strain in the suture between mild and moderate fusion. **(A)** micro-implant protraction device. **(B)** tooth-supported protraction device. **(C)** invisible aligner protraction device.

The experimental findings reveal that the peak equivalent elastic strain in the maxillary sutures varies among the three maxillary protraction devices (Figure 3). Notably, the extent of suture fusion influences the magnitude of the equivalent strain in the maxillary sutures during protraction but does not affect the stress distribution within these sutures. As the degree of suture fusion increases, the equivalent strain across all five sutures diminishes. At the same fusion level, the frontalmaxillary and nasofrontal suture experienced the highest forces in the micro-implant group models, whereas the zygomatic-temporal and palatine sutures endured the greatest forces in the invisible aligner group models. The variation in stress among the sutures was characterized by calculating the ratio of peak equivalent strain at mild fusion to that at moderate fusion for each observed suture within the same model. A higher ratio signifies a greater impact from the degree of suture fusion. For the tooth-supported anchorage group and micro-implant group, the pterygopalatine suture was the most affected by suture fusion, followed by the zygomatic temporal suture. In contrast, the Zygomaticomaxillary suture was the most affected in the invisible aligner group (Figure 4).

### 3.2 Equivalent stress distribution on the maxilla across different model groups

**FIGURE 5:**
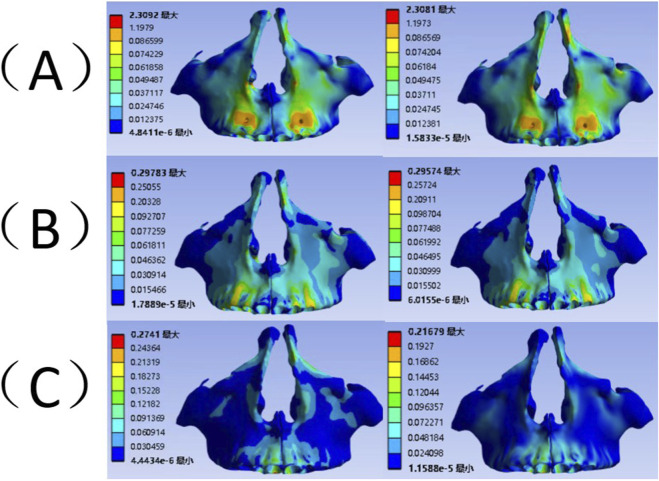
The equivalent stress distribution of maxilla. Left: Mild fusion. Right: Moderate fusion**(A)** The micro-implant group; **(B)**The tooth-supported; **(C)** The clear aligner group.

The distribution of equivalent stress in the maxilla varies considerably based on the type of orthodontic appliance used (Figure 5). When the degree of suture fusion is similar, the peak equivalent stress in the maxilla follows this order: micro-implant group, tooth anchorage group, and invisible aligner group. In the tooth anchorage group, elevated equivalent stress values were found at the bilateral maxillary canine abutments, with the highest stress occurring at the buccal gingival margin of the canines. Conversely, in the micro-implant group, significant stress values were observed at the implant sites and the maxillary frontal process, peaking at the micro-implant insertion points. The invisible aligner group displayed heightened equivalent stress at both the maxillary alveolar process and the frontal process, with the peak stress also occurring at the maxillary frontal process. Experimental findings suggest that the bone fusion has little effect on the equivalent stress values in the maxilla.

### 3.3 Displacement of craniofacial skeletal landmarks across model groups

At equivalent levels of sutural fusion, the invisible aligner group showed greater displacement of all skeletal landmarks compared to the other two groups, while the U1 point in the micro-implant group exhibited minimal displacement (Table 3). As sutural fusion advanced, maxillary protraction led to a reduction in craniofacial skeletal displacement across all three appliance types, although maxillary rotation increased. In the sagittal plane (Table 3), all landmarks across the three groups experienced anterior displacement. Specifically, in the tooth anchorage group, displacement magnitudes were as follows: U1 > A > ANS > PNS > OR > JU > N. In the micro-implant group, the order was ANS > A > PNS > U1 > OR > JU > N. For the invisible aligner group, the sequence was ANS > PNS > U1 > A > OR > JU > N. In the vertical plane (Table 4), all bony landmarks in the micro-implant group demonstrated downward displacement. Both the tooth anchorage and invisible aligner groups exhibited downward displacement for all landmarks except point N, which showed upward displacement. Furthermore, in both the mini-implant and tooth anchorage groups, the displacement of ANS was greater than that of PNS, indicating a clockwise rotation of the maxilla. However, regardless of the degree of sutural fusion, the vertical displacement of ANS in the invisible aligner group was less than that of PNS, suggesting a counterclockwise rotation of the maxilla.

**TABLE 3:** Displacements of landmarks in the Y-axis direction (μm).

Model number	Landmarks						
	ANS	A	N	U1	JU	OR	PNS
A1	7.097e-1	7.168e-1	3.079e-3	5.629e-1	2.727e-1	4.079e-1	6.702e-1
A2	5.017e-1	4.948e-1	4.808e-2	4.049e-1	2.079e-1	2.562e-1	4.489e-1
B1	6.203e-1	6.215e-1	−2.318e-2	1.240	2.450–1	3.341e-1	5.922e-1
B2	4.654e-1	4.676e-1	1.457e-2	1.080	1.982e-1	2.340e-1	4.364e-1
C1	2.489	2.349	−6.401e-2	2.414	7.065e-1	1.172	2.473
C2	1.133	1.054	−3.770e-2	9.680e-1	3.700e-1	5.506e-1	1.106

Y-axis, a negative value denotes posterior movement.

**TABLE 4:** Displacements of landmarks in the Z-axis direction (μm).

Model number	Landmarks						
	ANS	A	N	U1	JU	OR	PNS
A1	−3.048e-1	−3.052e-1	−1.623e-2	−4.276e-1	−2.063e-1	−1.514e-1	−1.981e-1
A2	−1.981e-1	−1.976e-1	−6.615e-3	−3.206e-1	−1.341e-1	−6.998e-2	−6.301e-2
B1	−1.894e-1	−1.873e-1	4.109e-2	−2.961e-1	−1.587e-1	−5.634e-2	−1.851e-1
B2	−1.296e-1	−1.270e-1	1.615e-2	−2.402e-1	−1.195e-1	−2.029e-2	−9.980e-2
C1	−6.308e-1	−5.958e-1	1.113e-2	−19.564	−1.018	−6.227e-1	−1.998
C2	−1.134e-1	−9.087e-2	4.900e-2	−19.091	−4.359e-1	−1.510e-1	−8.214e-1

Z-axis, a negative value denotes downward movement.

### 3.4 Three-dimensional displacement of maxillary incisors and first molars in each model group

As the degree of bone fusion increased, the displacement of incisors and first molars slightly decreased across all models, while the displacement trend remained unchanged (Table 5, Table 6). At equivalent levels of bone fusion, the total tooth displacement decreased in the following order: invisible aligner group, tooth anchorage group, and micro-implant group. In the sagittal plane (Table 5), all conditions showed a tendency for the incisor crowns to tip labially and the molar crowns to shift mesially. In the micro-implant and invisible aligner group, displacement progressively increased from anterior to posterior positions. Within the tooth-supported anchorage groups, the central incisors exhibited the greatest sagittal displacement, whereas the lateral incisors showed the smallest. In the vertical plane (Table 6), both incisors and molars demonstrated a tendency for extrusion under all conditions. For the micro-implant and invisible aligner groups, vertical displacement decreased progressively from anterior to posterior. Among the tooth-supported anchorage groups, the lateral incisors exhibited the largest vertical displacement, while the first molars showed the smallest.

**TABLE 5:** Displacements of landmarks in the Y-axis direction (μm).

Landmarks	Model number					
	A1	A2	B1	B2	C1	C2
a1	6.273e-1	4.006e-1	9.942e-1	8.235e-1	4.756	3.206
a2	6.583e-1	4.091e-1	4.143e-1	2.089e-1	6.004	4.369
a6	7.427e-1	4.643e-1	6.834e-1	4.730e-1	9.861	7.987

**TABLE 6:** Displacements of landmarks in the Z-axis direction (μm).

Landmarks	Model number					
	A1	A2	B1	B2	C1	C2
a1	−4.128e-1	−3.237e-1	−6.062e-1	−5.557e-1	−18.993	−18.590
a2	−3.799e-1	−3.033e-1	−1.316	−1.295	−12.461	−11.833
a6	−2.500e-1	−1.567e-1	−2.909e-1	−2.496e-1	−9.088e-1	−3.199e-1

Z-axis,a negative value represents tooth eruption.

## 4 Discussion

Anterior maxillary traction is a widely used approach for addressing sagittal maxillary hypoplasia. This technique applies mechanical stress to the maxillary suture, stimulating mesenchymal stem cells to promote new bone proliferation, deposition, and remodeling, which facilitates anterior and inferior growth of the maxilla. This biological mechanism underpins the histological rationale for the orthodontic technique (Liang et al., 2022). Recently, there has been ongoing debate about the effectiveness of maxillary anterior traction in children during their late growth phases (Hamidaddin, 2023). Some researchers assert that the ideal treatment window for this approach is before the age of 9 (Wendl et al., 2017), arguing that children in later growth stages experience only minimal skeletal effects from maxillary protraction and should therefore avoid this treatment. Conversely, other studies (Li et al., 2024) suggest that maxillary protraction can still advance the maxilla and enhance midface concavity in post-growth children. To investigate this further, the present study utilized three-dimensional finite element methods to create two representative craniofacial complex models with varying degrees of sutural fusion. It simulated commonly observed traction directions and force values to analyze the biomechanical effects on maxillary sutures, craniofacial bones, and teeth during maxillary protraction in children with differing levels of sutural fusion. Recent findings (Lee et al., 2025) indicate that the “track effect” and “buccal muscle mechanism” associated with the maxillary dentition make arch expansion therapy during maxillary protraction inadvisable for children with maxillary sagittal hypoplasia whose skeletal width variation falls within the normal range. Consequently, arch expansion factors were excluded from this study.

Numerous studies (Li et al., 2025b; Li et al., 2024; Hollander et al., 2022) have established that maxillary protraction therapy effectively promotes maxillary advancement and significantly enhances midface concavity, as well as the sagittal relationships between the maxilla and mandible, even in patients with skeletal Class III malocclusion who are beyond their peak growth phase. This research revealed that as the fusion of the bone sutures progresses, the displacement of the jawbones, and the degree of tooth movement all diminish. Notably, the sequence of applying forces to the sutures, the order of jawbone displacement, and the direction of tooth movement remained consistent throughout. These results suggest that the orthodontic effectiveness of maxillary anterior traction is primarily influenced by the anchorage design of the appliance. Although the level of sutural fusion affects the degree of facial improvement, it does not change the overall trend of enhancement. Additionally, the study observed that children with higher rates of suture fusion displayed an increase in compensatory tooth movement. Therefore, when conducting maxillary protraction treatment for patients in the later stages of growth, it is crucial to implement clinical strategies to control undesirable tooth movement in order to achieve optimal orthodontic results.

The experimental results indicate that the distribution of forces and stress levels within the maxillary suture varies significantly based on the type of maxillary protraction appliance utilized. This variation likely stems from differences in force transmission and anchorage design among the appliances. In this study, micro-implants were strategically placed between the roots of the maxillary canines and lateral incisors, reflecting clinical practice. The application point of the force was positioned higher than in the other two appliance groups, applying direct pressure to the maxillary bone, which resulted in enhanced new bone formation in the upper third of the facial structure. Conversely, the maxillary protraction device associated with the invisible aligner, featuring an angel hook situated between the crowns of the maxillary canine and lateral incisor, encompasses the entire dentition. This design facilitates a comprehensive sagittal forward displacement of the entire dentition, significantly encouraging new bone formation in the middle third of the face. During the use of clear aligners for maxillary protraction, the appliances experience deformation due to the forward traction forces. This results in a tendency for the anterior region to “widen and straighten,” leading to an expansion of the anterior dental arch. The experimental findings reveal that the median palatine suture in the invisible aligner was subjected to significantly greater force than in the other two groups. This phenomenon may be attributed to the expansion of the anterior dental arch that occurs during the forward traction process associated with the invisible aligner. Additionally, the study identified substantial forces acting on the pterygopalatine, frontonasal, and zygomaticotemporal sutures during maxillary protraction with all three types of appliances. Therefore, it is hypothesized that these three sutures play a crucial role in the sagittal anterior displacement of the maxilla, consistent with the conclusions drawn by Luo (2013). This study revealed that patients with comparable sutural fusion experience higher stress levels at the maxillary micro-implant insertion site, zygomatic process, and nasomaxillary suture during maxillary protraction with micro-implants. Notably, the peak equivalent stress was predominantly situated at the micro-implant insertion site, consistent with findings from previous research (Liu et al., 2015). Under similar conditions, the peak equivalent stress in the maxilla for the micro-implant group was found to be approximately 10–40 times greater than that observed in the other two groups. Consequently, when treating children with thin and low-density labial cortical bone, it is crucial to exercise caution against applying excessive traction forces during maxillary protraction to avoid potential loosening or dislodgment of the micro-implants. In cases involving maxillary protraction with a tooth-supported appliance, the equivalent stress in the maxilla primarily concentrated at the canine abutment. This suggests that traction forces can be transmitted upward along the canine abutment to other facial structures, indirectly indicating that the frontal and zygomatic sutures endure greater stress compared to other facial sutures when tooth-supported appliances are used. When comparing the first two maxillary protraction devices, the equivalent stress on the maxilla during traction with the invisible aligner traction device is primarily focused at the inferior margin of the foramen lacrimae and the lateral wall of the orbital floor. This finding suggests that the invisible aligner device exerts a distinct mechanical effect on the middle third of the facial bone tissue.

The invisible aligner differs from the other two types of aligners in terms of anchorage sources and force transmission mechanisms. The micro-implant anterior traction device uses the maxilla as an anchorage, effectively promoting maxillary movement while minimizing dental side effects, such as incisor labial tipping and molar extrusion. In contrast, tooth-supported anterior traction devices rely on adhesive or mechanical retention on the abutment teeth to provide counterforce, transmitting traction through the teeth to the maxilla and consequently inducing unwanted tooth movement. When combined with invisible aligners, anterior traction forces are transmitted through the polymer membrane to the teeth and subsequently to the maxilla. Due to the elastic properties of the polymer membrane, it undergoes a certain degree of deformation under anterior traction forces. This deformation generates a lateral component, resulting in an arch-widening effect concurrent with sagittal forward movement of the dentition. Furthermore, because the anterior traction component of the invisible aligner encases the crowns of the maxillary teeth and utilizes the entire maxillary dentition as anchorage, compensatory tooth movement during anterior traction is more pronounced than with the other two appliance types.

Patients with skeletal Class III malocclusion frequently exhibit facial concerns such as midface depression and nasal root collapse (Zhou et al., 2025). A thorough understanding of how various maxillary protraction devices influence facial bone movement is essential for effective treatment planning. In this study, the greatest sagittal displacement of skeletal landmarks was observed during maxillary protraction using clear aligners. This phenomenon may be attributed to increased stress on the zygomatic-temporal suture during this method, resulting in more significant movement of the zygomatic and maxillary bones. Hirato. (1984) reported that the center of resistance in the maxilla is located between the first and second premolars. When forces are applied below the center of resistance, the maxilla undergoes counterclockwise rotation; when forces are applied above the center of resistance, the maxilla undergoes clockwise rotation. Liu et al. (2015) demonstrated that when the anterior traction force point is applied between the maxillary canine and first molar crowns, the maxilla undergoes counterclockwise rotation. When the force point is shifted upward to the region between the maxillary canine and first molar roots, the maxilla undergoes clockwise rotation. The findings from this experiment indicate that, under the same force and direction of maxillary protraction, the maxilla rotated clockwise in both the micro-implant group and the tooth-supported maxillary protraction group, while a counterclockwise rotation was noted in the invisible aligner. This discrepancy may stem from the positioning of the traction hook. In this research, the micro-implant sites were strategically placed between the roots of the bilateral lateral incisors and canines. The traction hook on the tooth-supported maxillary protraction device was positioned at the mesial cervical gingival margin of the canine, whereas the traction clip for the invisible aligner maxillary protraction device was located at the crown junction between the lateral incisor and canine, which is significantly more occlusally positioned than the other two devices. Additionally, in the clear aligner group, since the traction force first acts on the membrane, the aligner deforms during anterior traction. This generates unnecessary lateral component forces, exerting a certain influence on the three-dimensional displacement of the maxilla. Consequently, for patients who do not wish their mandible to rotate clockwise, incorporating a bite block is advisable to prevent the mandible from rotating.

Anterior maxillary traction treatment is known to promote skeletal remodeling while simultaneously causing dental compensatory changes, such as the mesial movement of maxillary teeth, molar elongation, and the labial inclination of incisors (Baik et al., 2023). Consistent with findings from previous studies (Zhang et al., 2025a; Ma et al., 2024), this experiment demonstrated that maxillary protraction using all three appliances resulted in the elongation of both maxillary incisors and molars. Consequently, for patients exhibiting clinically high mandibular angles, incorporating a bite block is advisable to prevent clockwise mandibular rotation. To assess dental compensation, the displacement difference between point A and the incisors was calculated, with larger differences signifying greater dental compensation. The results indicated that clear aligners produced the most pronounced dental effects. Thus, it is recommended that clinicians utilizing clear aligners for maxillary protraction extend the marginal length of the appliances in the anterior region or refine attachment designs to mitigate the labial inclination of the incisors.

Clinically, families of children with skeletal Class III malocclusion often prefer non-surgical camouflage orthodontic treatments. However, research on this topic remains limited both nationally and internationally. Therefore, it is essential to understand the biomechanical mechanisms involved in maxillary protraction for children with lower growth potential. Previous studies have demonstrated that patients with varying degrees of sutural fusion experience significantly different clinical outcomes during orthodontic treatment (Angelieri et al., 2017a; Mao et al., 2003). In response, this study developed three-dimensional models of the craniofacial complex with differing elastic moduli of the sutures to simulate two typical degrees of maxillary suture fusion. By analyzing the effects of maxillary anterior traction under various loading conditions, this research offers biomechanical insights that can inform personalized treatment plans for patients at different growth and developmental stages. Additionally, it enhances orthodontists’ understanding of the mechanisms behind tooth-supported maxillary protraction appliances, micro-implant-assisted maxillary protraction appliances, and invisible aligner-based maxillary protraction appliances.

However, this implementation still has the following limitations. Three-dimensional finite element analysis, as a highly accurate and non-invasive method for studying oral biomechanics, is widely applied in orthodontics. However, it can only reflect the initial displacement and stress conditions of the craniofacial complex under a single applied load, and cannot reveal the actual bone formation that occurs after prolonged loading. Additionally, this experiment primarily assessed incisor compensation by evaluating the linear displacement difference between point A and the incisor landmark, without considering clinically relevant indicators such as tooth inclination angle. This approach has certain limitations, and subsequent clinical trials will be conducted to validate these experimental findings.

## 5 Conclusion

The findings of this study reveal that the degree of sutural fusion does not affect the stress distribution pattern or the overall trend of maxillary displacement during maxillary protraction; rather, it reduces the overall effectiveness of orthodontic treatment. Maxillary protraction appliances significantly influence facial growth patterns, with micro-implant anchors proving more effective in correcting nasal root depression, while invisible aligners are more successful in improving mid-face depression. This study provides biomechanical evidence that supports the creation of tailored treatment plans for children with maxillary sagittal hypoplasia.

## Data Availability

All relevant data is contained within the article: The original contributions presented in the study are included in the article/Supplementary Material, further inquiries can be directed to the corresponding author.
