# Supplementary material for: A finite element study on three maxillary protraction devices for treating maxillary sagittal hypoplasia at different levels of bone fusion
Source: Front Bioeng Biotechnol. 2026 Feb 11;14:1745618. doi: 10.3389/fbioe.2026.1745618 (PMC12932591; doi:10.3389/fbioe.2026.1745618)
Supplement: Supplementary file 1 [file Table1.docx]

Supplementary Material

# 1.Supplementary Figures and Tables

For more information on Supplementary Material and for details on the different file types accepted, please see [here](https://www.frontiersin.org/guidelines/author-guidelines#supplementary-material).

## Supplementary Tables

Ratio of the peak equivalent strain in the suture between mild and moderate fusion.

A： tooth-supported protraction device.B:micro-implant protraction device.C: invisible aligner protraction device

|  | Zygomaticomaxillary suture | Zygomatic temporal suture | median palatal suture | pterygopalatine suture | frontalmaxillary and nasomaxillary sutures |
| --- | --- | --- | --- | --- | --- |
| A | 3.42 | 4.142 | 3.225 | 4.17 | 3.77 |
| B | 3.81 | 3.939 | 2.363 | 5.303 | 3.29266 |
| C | 4.3 | 3.839 | 3.865 | 3.803 | 4.0507 |

Peak equivalent elastic strain in the five maxillary sutures under loading across different model

|  | ZMS | ZTS | MPS | PMS | FMS |
| --- | --- | --- | --- | --- | --- |
| A1 | 6.38E-04 | 3.61E-04 | 1.70E-04 | 2.21E-03 | 7.13E-04 |
| A2 | 1.87E-04 | 8.72E-05 | 5.28E-05 | 5.30E-04 | 1.89E-04 |
| B1 | 4.53E-04 | 2.94E-04 | 1.44E-04 | 1.52E-03 | 5.70E-04 |
| B2 | 1.19E-04 | 7.47E-05 | 6.10E-05 | 2.87E-04 | 1.73E-04 |
| C1 | 2.03E-04 | 4.00E-04 | 3.56E-04 | 7.80E-04 | 3.52E-04 |
| C2 | 4.72E-05 | 1.04E-04 | 9.22E-05 | 2.05E-04 | 8.70E-05 |
